# Fruit and vegetable consumption is associated with lower prevalence of asymptomatic diverticulosis: a cross-sectional colonoscopy-based study

**DOI:** 10.1186/s12876-020-01374-0

**Published:** 2020-07-11

**Authors:** Benjamin Maxner, Jessica McGoldrick, Danielle Bellavance, Po-Hong Liu, Ramnik J. Xavier, Joseph C. Yarze, Rocco Ricciardi, Kyle Staller, Daniel C. Chung, Hamed Khalili

**Affiliations:** 1 Division of Gastroenterology, Massachusetts General Hospital, Boston, MA USA; 2 Sidney Kimmel Medical College, Thomas Jefferson University, Philadelphia, PA USA; 3 Clinical and Translational Epidemiology Unit, Massachusetts General Hospital, Boston, MA USA; 4 Section of Colon and Rectal Surgery, Division of General and Gastrointestinal Surgery, Massachusetts General Hospital, Boston, MA USA; 5 Karolinska Clinical Epidemiology Unit, Karolinska Institutet, Solna, Sweden; 6 Digestive Healthcare Center- Crohn’s and Colitis Center, Massachusetts General Hospital, 165 Cambridge Street, 9th Floor, Boston, MA 02114 USA

**Keywords:** Diverticulosis, Diet, Fruit and vegetables, And epidemiology

## Abstract

**Background:**

Previous studies of the relationship between dietary factors and risk of diverticulosis have yielded inconsistent results. We therefore sought to investigate the association between consumption of fruit and vegetables and prevalent diverticulosis.

**Methods:**

Our study population included participants in the Gastrointestinal Disease and Endoscopy Registry (GIDER), a colonoscopy-based longitudinal cohort at the Massachusetts General Hospital, who provided comprehensive information on dietary intake and lifestyle factors using validated questionnaires prior to colonoscopy. Information on presence and location of diverticula was obtained from the endoscopist at the end of each procedure. We used Poisson regression modeling to calculate the prevalence ratios (PRs) and 95% confidence intervals (CIs).

**Results:**

Among 549 participants with a mean age of 61 years enrolled in GIDER, we confirmed diverticulosis in 245 (44.6%). The prevalence of diverticulosis appeared to decrease with higher consumption of fruit and vegetables (P_trend_ = 0.007 for fruit and 0.008 for vegetables, respectively). Compared to participants with less than five servings of vegetables per week, the multivariable-adjusted PRs of diverticulosis were 0.84 (95% CI, 0.60–1.17) with five to seven servings per week and 0.62 (95% CI, 0.44–0.89) with greater than one serving per day. Similarly, compared to participants with less than five servings per week of fruit, the multivariable-adjusted PR of diverticulosis was 0.60 (95% CI, 0.41–0.87) with greater than one serving per day. These associations were not modified by age, BMI, smoking, or red meat intake (All P_interaction_ > 0.055).

**Conclusion:**

In a colonoscopy-based longitudinal cohort study, we show that higher consumption of fruit and vegetables is associated with lower risk of prevalent diverticulosis.

## Background

In the United States, nearly a third of adults older than 50 years have diverticulosis [1], of whom around 4% will develop complications such as diverticulitis or diverticular bleeding [2, 3]. Together, these complications resulted in over 216,000 admissions, nearly 2.7 million ambulatory visits, and around 2.2 billion dollars in inpatient costs in 2012 [4].

Despite the increasing prevalence of diverticulosis and the potential for costly complications, the role of environmental factors in the pathogenesis of the disease remains poorly understood. Prior studies investigating dietary intake, specifically of fruit and vegetables, and risk of diverticulosis have yielded conflicting results. Early studies have suggested a protective role for dietary fiber in the development of diverticula [5–7], while more recent studies have found either positive or no associations between fruit and vegetables and total fiber intake and the prevalence of diverticulosis [8–11]. However, many of these results were based on dietary evaluations conducted after the detection of diverticula, either radiographically or by colonoscopy [6–9], or were conducted in Asia where there are significant differences in the prevalence and location of diverticulosis, limiting the generalizability of these studies to the western population [10, 11]. We therefore used dietary data in the Gastrointestinal Disease and Endoscopy Registry (GIDER) study to investigate the associations between fruit and vegetables consumption and the prevalence of diverticulosis.

## Methods

### Study population

We drew our study population from GIDER, a colonoscopy-based longitudinal cohort at the Massachusetts General Hospital (MGH). Participants older than 18 undergoing a screening or surveillance colonoscopy at MGH were invited to complete a comprehensive dietary, lifestyle, and medical history questionnaire before their colonoscopy. Patients with a history of gastrointestinal cancer, hereditary non-polyposis colorectal cancer, familial adenomatous polyposis, inflammatory bowel disease, or known bleeding disorders were excluded from enrollment in the cohort. In addition, patients who had used oral or intravenous antibiotics in the 2 months prior to their procedure were excluded. The study was approved by the Institutional Review Board at the Partners Human Research Committee. All recruitments were carried out in accordance with the Institutional Review Board regulations, with participants signing an informed consent form prior to data and sample collection.

### Evaluation of dietary intake

We collected data on participants’ dietary intake through the administered baseline questionnaire. Specifically, we used the Semiquantitative Food Frequency Questionnaire (SFFQ) to define categories of dietary intake and aggregated individual foods into broader food groups (Supplementary Table 1). Prior to the colonoscopy, participants were asked to report their weekly frequency of consumption of red meat, processed meats, white meat, shellfish, fish, dairy, starches, fruit, and vegetables on an eight-category scale (Never, 1 per week, 2–4 per week, 5–6 per week, 1 per day, 2–3 per day, 4–5 per day, 6+ per day). We focused our analysis on the consumption of fruit (apples, raisins, bananas, oranges, strawberries, blueberries, etc.), vegetables (salad, tomatoes, onions, greens, carrots, peppers, etc.), red meat (beef, hamburger, pork, lamb), and processed meats (sandwich meat, ham, salami, bologna, sausage, bacon, “hotdogs”). We examined the frequency of participants’ responses in each of the eight categories for these foods and combined the individual categories into tertiles of weekly consumption. We grouped fruit and vegetables intake into less than five times per week, five to seven times per week, and greater than once per day and further categorized red and processed meat intake into never or not in the last week, once per week, and greater than or equal to 2 times per week.

In addition to our brief dietary questionnaire, a subset of participants also completed the previously validated SFFQ [12]. In a validation study of 97 participants who completed both the short dietary questionnaire and SFFQ, the intraclass correlation coefficients for weekly consumption of fruit and vegetables were 0.55 and 0.52, respectively.

### Other variables

Participants’ body mass index (BMI) was calculated from their reported height and weight at baseline. We assessed physical activity by asking participants about the average time spent per week in the last year on various recreational activities using a previously validated physical activity questionnaire [13]. We then assigned a metabolic equivalent task (MET) to each activity based on previously established guidelines [14] and determined the average MET-hour/week for all activities. Smoking history was defined as current smokers or those who have smoked more than 100 cigarettes in the past and regular NSAID use was defined as greater than 2 tablets per week consistent with prior analyses [15, 16]. Bowel movement frequency was evaluated with the question, “How frequently do you have a bowel movement?” Participants were also asked about their dietary preferences with respect to red meat and were grouped into three patterns: Western standard diet (≥3 times/month), low-red-meat diet (< 3 times/month), and no-red-meat-diet.

### Outcome of interest

Our primary outcome of interest was the detection of colonic diverticulosis during the colonoscopy. A study coordinator obtained information on the presence and location of diverticula from the endoscopist at the end of each procedure.

### Statistical analyses

Participants with missing data on dietary intake, BMI, and physical activity were excluded from all analyses (*n* = 161). Specifically, 549 participants had no missing information on dietary or lifestyle data and were therefore eligible for our analyses. There were no significant differences in age (61.3 vs 61.4 years, *P* = 0.954), sex (46.6% vs 47.2% male, *P* = 0.898), or prevalence of diverticulosis (44.6% vs 47.2%, *P* = 0.563) between eligible participants and those excluded due to missing data. We used Poisson regression modeling with a robust error variance to calculate the prevalence ratios (PRs) and 95% confidence intervals (CIs) while adjusting for age, sex, smoking, BMI, physical activity, dietary pattern, regular NSAID use, and number of bowel movements per day. We elected to use Poisson regression instead of logistic regression because the outcome of interest, diverticulosis, was common [17]. Additionally, we did attempt to use a log-binomial model, which uses a binomial distribution and have been shown to be slightly less biased than Poisson regression [18], but were unable to due to issues with model convergence. In addition, we evaluated for effect modification by age, BMI, smoking history, and red meat intake on associations between fruit and vegetables consumption and prevalence of diverticulosis by including cross-product terms of these potential risk factors and vegetables and fruit intakes in the multivariable models. All *P*-values were 2-sided and *P* < 0.05 were considered statistically significant. We used R version 3.2.0 for all analyses.

## Results

We confirmed diverticulosis in 245 participants (44.6%) among 549 enrolled in the GI Disease and Endoscopy Registry with a mean age of 61 years. Participants with diverticulosis were more likely to be older, male, smokers, and have a higher BMI than those without diverticula (Table 1). The rates of diverticulosis varied from 57% among those who consumed fruit and vegetables less than five times per week to 32% among those who consumed greater than one serving per day (Table 2). We found significant inverse associations between the intake of both fruit and vegetables and the prevalence of diverticulosis (P_trend_ = 0.007 for fruit and 0.008 for vegetables, respectively). Compared to participants with less than five servings of vegetables per week, the multivariable-adjusted PRs of diverticulosis were 0.84 (95% CI, 0.60–1.17) with five to seven servings per week and 0.62 (95%, 0.44–0.89) with greater than one serving per day. Similarly, we observed a lower prevalence of diverticulosis with five to seven servings of fruit per week (PR = 0.81; 95% CI, 0.58–1.11) and greater than one serving of fruit per day (PR = 0.60; 95% CI, 0.41–0.87) compared to participants with less than five servings per week even after adjusting for age, sex, BMI, smoking, physical activity, regular NSAID use, dietary pattern, and bowel movement frequency. We also created cross-classified categories of fruit and vegetables intake and observed that compared to individuals with less than or equal to one serving of fruit and vegetables per day, the multivariable-adjusted PR of diverticulosis was 0.58 (95% CI, 0.39–0.87) with greater than one serving per day.

**Table 1:** Baseline Characteristics of Participants According to Diagnosis of Diverticulosis

Characteristics	Mean ± SD or n(%)	
	No Diverticulosis ***n*** = 304	Confirmed Diverticulosis ***n*** = 245
Age, years	58.3 ± 9.6	65.1 ± 9.1
Gender, male	125 (41.1)	130 (53.1)
Body Mass Index (kg/m^2^)	26.4 ± 5.2	28.2 ± 5.8
Smoking History		
Current	7 (2.3)	9 (3.7)
Past	80 (26.3)	94 (38.4)
Never	213 (70.1)	139 (56.7)
Physical activity^a^, MET/week	40.3 ± 35.9	32.3 ± 37.1
Regular NSAID Use in Past 2 Years (> 2 tablets/week)	74 (24.3)	72 (30.2)
Dietary Pattern		
Western standard	164 (54.4)	141 (57.6)
Low red meat (< 3 times/month)	104 (34.2)	78 (31.8)
No red meat	23 (7.6)	23 (9.4)
Number of bowel movements		
> 1/day	61 (20.1)	31 (12.7)
1/day	151 (49.8)	135 (55.1)
< 1/day	86 (28.3)	76 (31.0)

^a^Physical activity includes jogging, running, biking, swimming, tennis, aerobics, and other vigorous exercises

*Abbreviation*: *MET* metabolic equivalent task

**Table 2:** Consumption of Fruit, and Vegetables and Prevalence of Diverticulosis

				*P* for trend
	< 5 times/week	5–7 times/week	> 1 time/day	
Vegetables				
Total subjects	126	160	215	
No. of cases, n (%)	72 (57.1)	82 (51.3)	69 (32.1)	
Age-adjusted	1.00	0.85 (0.62–1.17)	0.58 (0.42–0.81)	0.001
Model 2^a^	1.00	0.85 (0.61–1.19)	0.65 (0.46–0.92)	0.015
Model 3^b^	1.00	0.84 (0.60–1.17)	0.62 (0.44–0.89)	0.008
Fruits				
Total subjects	200	152	139	
No. of cases, n (%)	102 (51.0)	69 (45.4)	47 (33.8)	
Age-adjusted	1.00	0.83 (0.61–1.13)	0.61 (0.43–0.87)	0.006
Model 2^a^	1.00	0.82 (0.59–1.13)	0.64 (0.44–0.92)	0.015
Model 3^b^	1.00	0.81 (0.58–1.11)	0.60 (0.41–0.87)	0.007

NOTE. Prevalence ratio (95% CI) is shown unless otherwise indicated

^a^Adjusted for age (years), physical activity (MET-hours/week), BMI (kg/m^2^), sex, regular NSAID use (> 2 times/week), smoking (Current smoker, smoked in the past, never smoked), and dietary pattern (Western standard diet, low-red-meat diet, no-red-meat diet)

^b^Adjusted for age (years), physical activity (MET-hours/week), BMI (kg/m^2^), regular NSAID use (> 2 times/week), smoking (Current smoker, smoked in the past, never smoked), dietary pattern (Western standard diet, low-red-meat diet, no-red-meat diet), and number of bowel movements (< 1/day, 1/day, > 1/day)

We also explored the association between red and processed meat and prevalent diverticulosis. Compared to individuals who reported no consumption, the multivariable-adjusted PRs of diverticulosis with 2 or more servings per week were 1.03 (95% CI: 0.62–1.71) for red meat and 0.97 (95% CI 0.68–1.40) for processed meat, respectively.

Finally, we examined the associations between the consumption of fruit and vegetables and the prevalence of diverticulosis according to strata defined by potential risk factors and found no evidence for effect modification by age, BMI, smoking, and red meat intake (All P_interaction_ > 0.055) (Tables 3 and 4).

**Table 3:** Risk of diverticulosis according to vegetables consumption by strata

Stratum	< 5 times/week	5–7 times/week	> 1 time/day	P for interaction
**Vegetables**				
**Age**				0.128
*< 60 years*				
No. of cases	21/51	20/60	19/98	
Prevalence Ratio (95% CI)				
Age adjusted	1.00	0.81 (0.44–1.49)	0.45 (0.24–0.85)	
Multivariable-adjusted	1.00	0.81 (0.42–1.57)	0.53 (0.27–1.01)	
*≥60 years*				
No. of cases	51/75	62/100	50/117	
Prevalence Ratio (95% CI)				
Age adjusted	1.00	0.88 (0.61–1.28)	0.64 (0.43–0.95)	
Multivariable-adjusted	1.00	0.87 (0.59–1.29)	0.70 (0.46–1.05)	
**BMI (kg/m**^**2**^**)**				0.842
*< 25*				
No. of cases	20/42	22/50	25/95	
Prevalence Ratio (95% CI)				
Age adjusted	1.00	0.96 (0.53–1.76)	0.62 (0.34–1.12)	
Multivariable-adjusted	1.00	1.02 (0.54–1.92)	0.72 (0.39–1.34)	
*≥25*				
No. of cases	52/84	60/110	44/120	
Prevalence Ratio (95% CI)				
Age adjusted	1.00	0.82 (0.57–1.19)	0.60 (0.40–0.90)	
Multivariable-adjusted	1.00	0.82 (0.55–1.21)	0.65 (0.43–0.99)	
**Smoking**				0.778
*Current or past*				
No. of cases	27/40	31/42	19/58	
Prevalence Ratio (95% CI)				
Age adjusted	1.00	1.06 (0.65–1.72)	0.59 (0.35–0.99)	
Multivariable-adjusted	1.00	1.05 (0.63–1.75)	0.67 (0.39–1.16)	
*Never*				
No. of cases	33/63	32/79	29/103	
Prevalence Ratio (95% CI)				
Age adjusted	1.00	0.72 (0.47–1.11)	0.58 (0.37–0.89)	
Multivariable-adjusted	1.00	0.73 (0.47–1.13)	0.64 (0.41–1.01)	
**Red Meat Intake**				0.938
*< 2 times/week*				
No. of cases	27/46	23/40	17/68	
Prevalence Ratio (95% CI)				
Age adjusted	1.00	1.11 (0.68–1.80)	0.55 (0.33–0.94)	
Multivariable-adjusted	1.00	1.04 (0.63–1.74)	0.59 (0.34–1.02)	
*≥2 times/week*				
No. of cases	28/45	41/79	28/84	
Prevalence Ratio (95% CI)				
Age adjusted	1.00	0.66 (0.42–1.04)	0.56 (0.35–0.88)	
Multivariable-adjusted	1.00	0.71 (0.44–1.13)	0.64 (0.40–1.04)

**Table 4:** Risk of diverticulosis according to fruit consumption by strata

Stratum	< 5 times/week	5–7 times/week	> 1 time/day	P for interaction
**Fruit**				
**Age**				0.055
*< 60 years*				
No. of cases	35/93	15/60	7/51	
Prevalence Ratio (95% CI)				
Age adjusted	1.00	0.66 (0.36–1.21)	0.36 (0.16–0.80)	
Multivariable-adjusted	1.00	0.62 (0.33–1.15)	0.33 (0.14–0.81)	
*≥60 years*				
No. of cases	67/107	54/92	40/88	
Prevalence Ratio (95% CI)				
Age adjusted	1.00	0.92 (0.64–1.31)	0.72 (0.49–1.06)	
Multivariable-adjusted	1.00	0.92 (0.63–1.36)	0.77 (0.51–1.17)	
**BMI (kg/m**^**2**^**)**				0.317
*< 25*				
No. of cases	29/63	22/57	15/62	
Prevalence Ratio (95% CI)				
Age adjusted	1.00	0.75 (0.43–1.30)	0.49 (0.26–0.92)	
Multivariable-adjusted	1.00	0.75 (0.41–1.37)	0.57 (0.29–1.09)	
*≥25*				
No. of cases	73/137	47/95	32/77	
Prevalence Ratio (95% CI)				
Age adjusted	1.00	0.89 (0.61–1.28)	0.72 (0.47–1.09)	
Multivariable-adjusted	1.00	0.85 (0.58–1.26)	0.70 (0.45–1.09)	
**Smoking**				0.473
*Current or past*				
No. of cases	39/60	20/37	17/41	
Prevalence Ratio (95% CI)				
Age adjusted	1.00	0.95 (0.58–1.54)	0.69 (0.42–1.14)	
Multivariable-adjusted	1.00	0.91 (0.55–1.51)	0.72 (0.42–1.23)	
*Never*				
No. of cases	45/97	32/80	12/60	
Prevalence Ratio (95% CI)				
Age adjusted	1.00	0.77 (0.51–1.15)	0.55 (0.34–0.90)	
Multivariable-adjusted	1.00	0.74 (0.48–1.14)	0.58 (0.35–0.97)	
**Red Meat Intake**				0.925
*< 2 times/week*				
No. of cases	35/67	19/39	12/47	
Prevalence Ratio (95% CI)				
Age adjusted	1.00	0.92 (0.57–1.52)	0.65 (0.39–1.09)	
Multivariable-adjusted	1.00	0.83 (0.49–1.41)	0.68 (0.39–1.17)	
*≥2 times/week*				
No. of cases	45/79	31/75	18/52	
Prevalence Ratio (95% CI)				
Age adjusted	1.00	0.71 (0.47–1.08)	0.63 (0.39–1.02)	
Multivariable-adjusted	1.00	0.72 (0.47–1.12)	0.65 (0.39–1.09)	

## Discussion

In this colonoscopy-based cohort, fruit and vegetables consumption was significantly associated with a lower prevalence of diverticulosis even after adjusting for common risk factors. Conversely, no associations were observed between red and processed meat intake and the risk of prevalent diverticulosis. Furthermore, we did not observe any effect modification by age, BMI, smoking, or red meat intake on the association between fruit and vegetables intake and prevalent diverticulosis.

Our finding of a lower risk of prevalent diverticulosis among participants with a higher fruit and vegetables intake is supported by several prior studies. In a large cohort nested within the Health Professionals Follow-up Study, Aldoori and colleagues [19] found high-fiber diet to be protective against symptomatic diverticular disease. In a case-control study in Greece, Manousos et al.^6^ observed a significant inverse association between vegetables intake and prevalence of symptomatic diverticular disease. Similarly, a study of diet and diverticulosis restricted to asymptomatic patients found that vegetarians had a lower prevalence of diverticulosis compared to non-vegetarians [20]. However, these studies either focused on symptomatic diverticulosis [6, 19] or failed to fully account for other lifestyle and confounding factors [20]. Therefore, our study, which carefully adjusts for known and putative risk factors for diverticulosis, significantly extends these findings.

In contrast to our findings, two studies conducted in South Korea and Taiwan found no significant associations between the consumption of fruit and vegetables and risk of diverticulosis [10, 11]. However, the prevalence of diverticulosis in East Asian countries varies from 8 to 25%, an estimate that is significantly lower than those reported by us and others in Western populations [8–11, 20–22]. In addition, diverticulosis predominately affects the right colon in Asia compared to the left in the West, likely due to distinct risk factors and mechanisms of development [10]. In the U.S., a cross-sectional, colonoscopy-based study observed no associations between the intake of fiber from fruit and vegetables and risk of diverticulosis, while total fiber intake was found to be associated with a higher prevalence of diverticulosis [8]. Similarly, a more recent study by the same research group found no associations between the consumption of fruit and vegetables fiber and total fiber and the prevalence of diverticulosis [9]. However, dietary information was collected up to three to 4 months after colonoscopy in both studies and participants may have been aware of their diagnoses at the time of diet collection, increasing the likelihood of recall bias [8, 9]. Thus, misclassification of exposures may account for the lack of association between intake of fiber from fruit and vegetables and risk of prevalent diverticulosis.

The mechanism by which fruit and vegetables consumption influences the development of diverticulosis is currently unknown. Nearly fifty years ago, Painter postulated that low-fiber diets produce increased colonic pressures. Along with segmentation of the colon, these higher pressures cause the mucosa to herniate through weak areas in the muscle wall and form diverticula [5]. However, this hypothesis was based on ecological observations comparing the prevalence of diverticulosis in the West to that of native Africans, which failed to account for confounding variables and did not confirm the presence of diverticula in participants. The interaction between diet and the gut microbiota may mediate this process. In a recent study, Barbara et al. [23] observed a depletion of *Clostridium* cluster IV in the gut microbiota of asymptomatic diverticulosis cases compared to controls without diverticula. *Clostridium* cluster IV includes many anti-inflammatory bacterial species that primarily function through the release of butyrate, the preferred energy source for colonocytes and a key contributor to the integrity of the colonic epithelial barrier [24]. In turn, increased intake of fruit and vegetables has been shown to significantly increase the abundance of *Clostridium* cluster IV in the gut microbiome [25]. Thus, fruit and vegetables consumption through its effect on Clostridium cluster IV could decrease the risk of diverticula formation. Nevertheless, further studies are needed to better examine this complex relationship between diet, gut microbiota, and diverticulosis.

Our study has several strengths. First, all cases of diverticulosis were confirmed through complete colonoscopy. Second, our study population was drawn from a longitudinal cohort in which dietary and lifestyle data were collected prior to colonoscopy, averting the potential for recall bias. Third, we accounted for multiple risk factors that could impact our observed associations, including age, BMI, smoking history, bowel movement frequency, dietary pattern, regular NSAID use, and physical activity.

The present study has several potential weaknesses that are worth highlighting. First, the participants were from a single center and predominately white, which may reduce the generalizability of our observations. Specifically, out of 549 participants enrolled in the cohort, 511 (93.1%) identified as Caucasian, 19 identified as Asian (3.5%), 13 identified as African-American (2.4%), 3 (0.5%) identified as Pacific Islander or Native Hawaiian, and 3 (0.5%) identified as Native American. Second, we based our brief dietary questionnaire on the SFFQ; however, our validation showed a moderate to good correlation between the two measures. In addition, adjusting for measurement errors would likely strengthen our observed association. Third, our dietary questionnaire did not distinguish between different types of fruit and vegetables. Therefore, we were unable to evaluate the relationship between individual foods in these groups and the prevalence of diverticulosis. Lastly, although dietary data were collected prior to the colonoscopy, diverticula were likely present for many years, preventing us from demonstrating a clear temporal association between fruit and vegetables consumption and risk of diverticulosis. However, medium-term studies on patterns of dietary intake over time have demonstrated remarkable stability [26]. Additionally, some participants may have known about their diagnosis of diverticulosis from prior colonoscopies (~ 40% had 2 or more colonoscopies prior to enrollment).

## Conclusions

In conclusion, we found that frequent consumption of fruit and vegetables is associated with a decreased prevalence of colonic diverticulosis. Although our study does not address specific foods within these food groups, our results call to attention the potential role of diet as a whole in the prevention of colonic diverticula. Due to an aging population in the U.S., the prevalence and cost of diverticulosis are expected to continue to rise, highlighting the critical importance of identifying modifiable risk factors. Future studies should focus on the potential mechanisms underlying these associations, particularly with regards to the mediating effect of gut microbiome.

## Supplementary information

**Additional file 1: Supplementary Table 1.** Brief dietary questionnaire. Dietary assessment instrument administered to each participant to evaluate nutritional intake.

## Data Availability

The datasets generated and/or analyzed during the current study are not publicly available, but de-identified data may be available pending ethical committee approval.

## Abbreviations

GIDER: Gastrointestinal Disease and Endoscopy Registry
PR: Prevalence ratio
CI: Confidence interval
SFFQ: Semiquantitative Food Frequency Questionnaire
MET: Metabolic equivalent task
BMI: Body mass index
