# Supplementary material for: Fruit and vegetable consumption is associated with lower prevalence of asymptomatic diverticulosis: a cross-sectional colonoscopy-based study
Source: BMC Gastroenterol. 2020 Jul 11;20:221. doi: 10.1186/s12876-020-01374-0 (PMC7353809; doi:10.1186/s12876-020-01374-0)
Supplement: Supplementary file 1 — Additional file 1: Supplementary Table 1. Brief dietary questionnaire. Dietary assessment instrument administered to each participant to evaluate nutritional intake. [file 12876_2020_1374_MOESM1_ESM.docx]

|  | DID YOU EAT THIS PRODUCT IN THE LAST 24 HOURS? (Check if Yes) | HOW OFTEN DID YOU EAT OR DRINK THE FOLLOWING PRODUCTS IN THE ***LAST WEEK***? | | | | | | | |
| --- | --- | --- | --- | --- | --- | --- | --- | --- | --- |
|  |  | Never or not in the last week | 1 per week | 2-4 per week | 5-6 per week | 1 per day | 2-3 per day | 4-5 per day | 6+ per day |
| **Tea or coffee no sugar and no sugar replacement** |  |  |  |  |  |  |  |  |  |
| **Soft drinks, tea or coffee with sugar** (corn syrup, maple syrup, cane sugar, etc.) |  |  |  |  |  |  |  |  |  |
| **Diet soft drinks, tea or coffee with sugar substitute** (Stevia, Equal, Splenda, etc.) |  |  |  |  |  |  |  |  |  |
| **Fruit juice** (orange, apple, cranberry, prune, etc.) |  |  |  |  |  |  |  |  |  |
| **Water** |  |  |  |  |  |  |  |  |  |
| **Alcohol** (beer, brandy, spirits, hard liquor, wine, aperitif, etc.) |  |  |  |  |  |  |  |  |  |
| **Dairy** (milk, cream, ice cream, yogurt, cheese, cream cheese) |  |  |  |  |  |  |  |  |  |
| **Fruits (no juice)** (Apples, raisins, bananas, oranges, strawberries, blueberries, etc. (frozen or fresh) |  |  |  |  |  |  |  |  |  |
| **Vegetables** (salad, tomatoes, onions, greens, carrots, peppers, green beans, etc.) |  |  |  |  |  |  |  |  |  |
| **Beans** (tofu, soy, soy burgers, lentils, Mexican beans, lima beans, etc.) |  |  |  |  |  |  |  |  |  |
| **Starch** (bread, pizza, potatoes, yam, rice, wheat, cereals, pancakes, etc.) |  |  |  |  |  |  |  |  |  |
| **Eggs** |  |  |  |  |  |  |  |  |  |
| **Red meat** (beef, hamburger, pork, lamb) |  |  |  |  |  |  |  |  |  |
| **White meat** (chicken, turkey, etc.) |  |  |  |  |  |  |  |  |  |
| **Processed meats** (lunch meat, sandwich meat, ham, salami, bologna, sausage, kielbasa, hotdog, bacon, etc.) |  |  |  |  |  |  |  |  |  |
| **Shellfish** (shrimp, lobster scallops, etc.) |  |  |  |  |  |  |  |  |  |
| **Fish** (fish nuggets, breaded fish, fish cakes, salmon, tuna, etc.) |  |  |  |  |  |  |  |  |  |
| **Sweets** (pies, jam, chocolate, cake, cookies, etc.) |  |  |  |  |  |  |  |  |  |

**Supplementary Table 1: Brief dietary questionnaire**
